# Autophagy guards tendon homeostasis

**DOI:** 10.1038/s41419-022-04824-7

**Published:** 2022-04-23

**Authors:** Costanza Montagna, Rene B. Svensson, Monika L. Bayer, Salvatore Rizza, Emiliano Maiani, Ching-Yan Chloé Yeung, Giuseppe Filomeni, Michael Kjær

**Affiliations:** 1 Institute of Sports Medicine Copenhagen, Department of Orthopedic Surgery, Copenhagen University Hospital—Bispebjerg and Frederiksberg, Copenhagen, Denmark; 2 Center for Healthy Aging, Department of Clinical Medicine, University of Copenhagen, Copenhagen, Denmark; 3 Redox Signaling and Oxidative Stress Group, Danish Cancer Society Research Center, Copenhagen, Denmark; 4 Present Address: Unicamillus-Saint Camillus, University of Health Sciences, Rome, Italy; 5 Department of Biology, Tor Vergata University, Rome, Italy

**Keywords:** Physiology, Cell biology

## Abstract

Tendons are vital collagen-dense specialized connective tissues transducing the force from skeletal muscle to the bone, thus enabling movement of the human body. Tendon cells adjust matrix turnover in response to physiological tissue loading and pathological overloading (tendinopathy). Nevertheless, the regulation of tendon matrix quality control is still poorly understood and the pathogenesis of tendinopathy is presently unsolved. Autophagy, the major mechanism of degradation and recycling of cellular components, plays a fundamental role in the homeostasis of several tissues. Here, we investigate the contribution of autophagy to human tendons’ physiology, and we provide in vivo evidence that it is an active process in human tendon tissue. We show that selective autophagy of the endoplasmic reticulum (ER-phagy), regulates the secretion of type I procollagen (PC1), the major component of tendon extracellular matrix. Pharmacological activation of autophagy by inhibition of mTOR pathway alters the ultrastructural morphology of three-dimensional tissue-engineered tendons, shifting collagen fibrils size distribution. Moreover, autophagy induction negatively affects the biomechanical properties of the tissue-engineered tendons, causing a reduction in mechanical strength under tensile force. Overall, our results provide the first evidence that autophagy regulates tendon homeostasis by controlling PC1 quality control, thus potentially playing a role in the development of injured tendons.

## Introduction

Tendon is a dense collagenous connective tissue that transmits the force from contracting skeletal muscle to bone to ensure bodily movement, and its matrix is dominated by type I collagen (Col-I) [1, 2]. Although the tendon collagen matrix is primarily synthesized during childhood and adolescence [3], in adulthood, tendon cells increase collagen synthesis and turnover in response to physiological mechanical loading [4] and pathological overloading [5]. More recently, it has been demonstrated that there is a daily coordinated secretion of Col-I in murine tendon cells [6], and the ability of tendon cells to remodel the extracellular matrix (ECM) has been suggested to be a risk factor for tendon pathologies, such as tendinopathy [7–11]. However, the molecular mechanism that underlies the development of tendinopathy remains an enigma.

Autophagy is an early cellular response to stress, crucial for the elimination of unwanted proteins and organelles during tissue structure remodeling. Autophagy plays a pivotal role in tissue homeostasis [12, 13] by mediating the degradation and recycling of cellular components, which are engulfed in vesicles (autophagosomes) and dismantled upon fusion with lysosomes. Procollagens, the precursors of collagen molecules, are the most abundant gene products in multicellular animals. In particular, type I procollagen (PC1) consists of two pro-α1 and one pro-α2 chain that is folded into a triple-helical protein in the ER. The process of procollagen folding is complex and, therefore, a portion of newly synthesized procollagen gets usually misfolded [14]. To prevent the accumulation of excess collagen in the ER, autophagy is able to degrade portions of the ER containing procollagen aggregates through different ER-phagy pathways [15–18]. Indeed, ectopic procollagen accumulates at specific ER subdomains, the ER exit sites (ERES), and can be directly engulfed by lysosomes via non-canonical micro-ER-phagy [16, 17]. However, a more selective mechanism for misfolded procollagen recognition has been described [18, 19]. In this case, the ER-resident chaperone calnexin recognizes luminal misfolded procollagens and interacts with the ER-phagy receptor RETREG1/FAM134B. RETREG1/FAM134B harbors a functional LC3-interacting region (LIR), which mediates binding to autophagosomes and delivers the portion of the ER containing both calnexin and procollagen to the lysosome for degradation [18]. The present study provides the proof of concept that autophagy is an active mechanism in tendons and is essential for procollagen turnover. The involvement of autophagy in tendon homeostasis provides the first evidence for a new field of translational research, bridging the gap between physiologically healthy tendon biology, adaptation to loading, and the development of tendon pathology.

## Results

### Autophagy is active in tendon tissues and promotes the degradation of intracellular procollagen 1

**Fig. 1:**
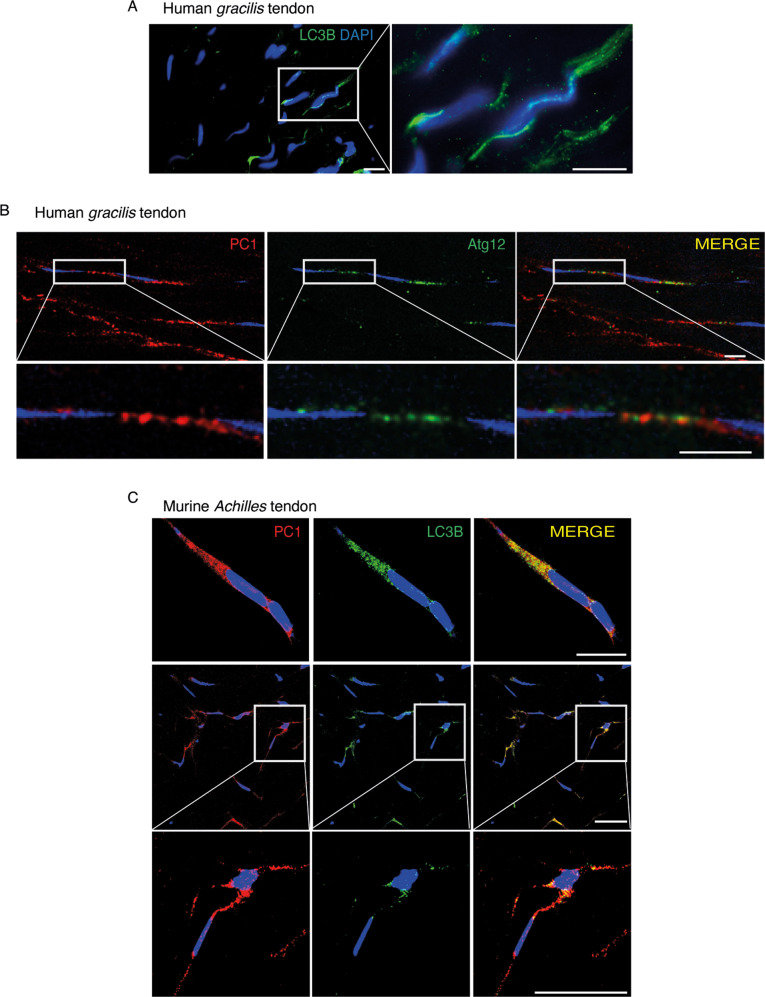
Autophagy degrades PC1 in tendons. **A** Representative images of LC3B puncta (autophagosomes) in human *gracilis* tendon. Human tendon tissues were immunolabeled for LC3B (green), nuclei stained with DAPI (blue) and analyzed by confocal microscopy. Scale bar = 10 μm. Inset shows a higher magnification of selected areas. **B** Representative images of human *gracilis* tendon immunolabeled for PC1 (red), Atg12 (green) and nuclei stained with DAPI (blue). Tissues were analyzed by confocal microscopy. Scale bars = 10 μm. The insets show higher magnification and single color channels of the boxed areas. **C** Representative images of murine *Achilles* tendons immunolabeled for PC1 (red), LC3B (green) and nuclei stained with DAPI (blue). Tissues were analyzed by confocal microscopy. Scale bars = 10 μm. The insets show higher magnification and single color channels of the boxed areas.

With the aim of understanding whether autophagy is an active process in tendons, we made use of human tendon samples and performed immunofluorescence analyses to detect autophagosomes structures. In human *gracilis* tendons (Fig. S1A), we observed, for the first time, the presence of LC3B-positive puncta (Fig. 1A), which are commonly assumed to be autophagic vesicles. Moreover, these tendons show Atg12-positive autophagic vesicles (AVs) that localized in proximity to PC1 (Figs. 1B and S1B, C) (Pearson’s coefficient: 0.457 ± 0,168), suggesting that PC1 could be targeted by autophagy for degradation. This hypothesis was supported by further observations in murine *Achilles* tendons (Fig. S1D), where we detected a strong colocalization between LC3B and PC1 (Figs. 1C and S1E, F) (Pearson’s coefficient: 0.7397 ± 0,112). This evidence confirmed that PC1 was retained in autophagosomes and suggested that autophagy could play a role in procollagen processing. Interestingly, the number of AVs seemed to increase at the myotendinous junctions of both human *gracilis* and murine *Achilles* tendons (Fig. S1G–J).

**Fig. 2:**
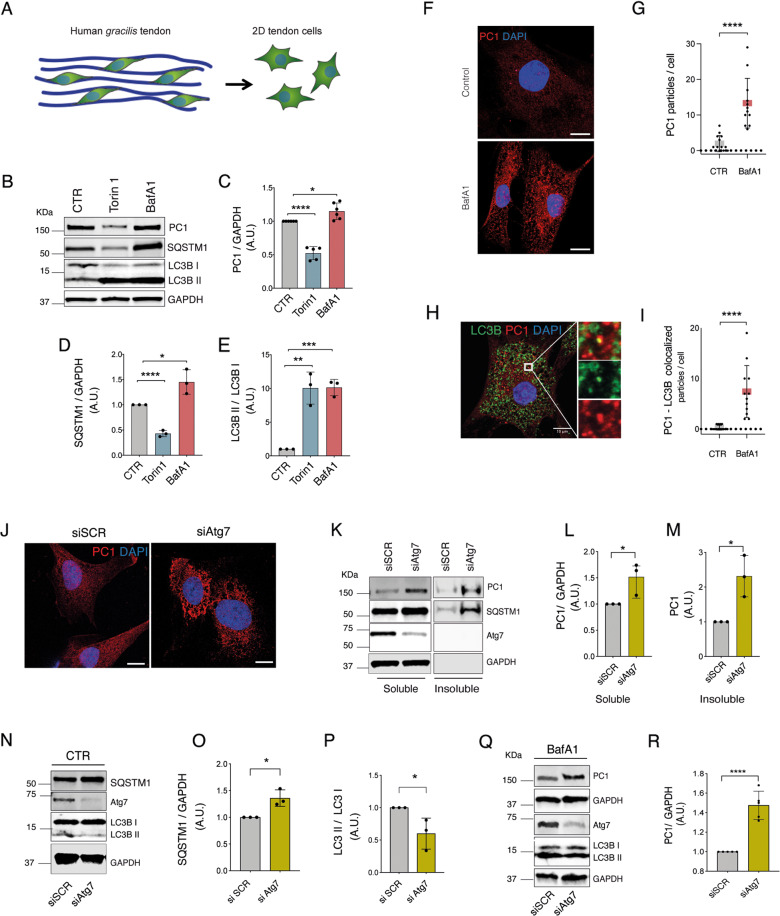
PC1 is an autophagy substrate in primary human tendon cells. **A** Graphical representation of cells isolated from *gracilis* tendons harvested from patients that underwent reconstructive anterior cruciate ligament surgery. **B** Western blot analysis of PC1, LC3B, and SQSTM1/p62 in Control, Torin 1 and Bafilomycin A1 (BafA1) treated cells for 4 h. GAPDH was used as a loading control. Quantification of the PC1 (**C**), SQSTM1/p62 proteins level (**D**) LC3BII/LC3BI ratio (**E**) western blot data. Data are representative of five (PC1) or three (SQSTM1/p62 and LC3BII/LC3BI) independent experiments made with cells from five or three different human donors. *p < 0.05, ****p < 0.0001, unpaired *t*-test. **F** Human tendon cells control and treated with BafA1 for 16 h were immunolabeled for PC1 (red), nuclei stained with DAPI (blue) and analyzed by confocal microscopy. Scale bar = 10 μm. **G** Quantification of PC1 particles per cell. ****p < 0.0001, unpaired *t*-test. **H** Cells treated with BafA1 for 16 h were immunolabeled for PC1 (red), LC3B (green) and nuclei stained with DAPI (blue). Scale bars = 10 μm. The insets show higher magnification and single color channels of the boxed area. **I** Quantification of PC1-LC3B colocalized particles per cell. ****p < 0.0001, unpaired *t*-test. **J** Control cells treated with scrambled siRNA (siSCR) of siAtg7 siRNAs were immunolabeled for PC1 (red), nuclei stained with DAPI (blue) and analyzed by confocal microscopy. Scale bar = 10 μm. **K** Western blot analysis of PC1, SQSTM1/p62, and Atg7 in control (siSCR) and siAtg7 siRNA-treated cells. Cell lysates were separated into soluble and insoluble fractions. GAPDH was used as a loading control. Quantification of normalized PC1 protein level in soluble (**L**) and insoluble (**M**) fractions. Data are representative of three independent experiments made with cells from three different human donors. *p < 0.05, unpaired *t*-test. **N** Western blot analysis of SQSTM1/p62, Atg7, and LC3B in siSCR and siAtg7 cells. GAPDH was used as a loading control. Quantification of SQSTM1/p62 (**O**) and LC3BII/LC3BI ratio (**P**). **Q** Western blot analysis of PC1 Col-I, Atg7, and LC3B in siSCR and siAtg7 cells treated with BafA1. **R** Quantification of the normalized PC1 Col-I protein. Data are representative of five independent experiments made with cells from five different human donors. ****p < 0.0001, unpaired *t*-test.

In order to investigate whether autophagy affects tendon PC1 levels, we isolated and cultured primary human tendon cells from the human *gracilis* tendon (Fig. 2A). Once put in culture, autophagy was activated by Torin 1, or inhibited by the lysosomal acidification inhibitor Bafilomycin 1 (BafA1). Then, autophagy flux was monitored by western blot analysis of LC3B (i.e., the conversion of LC3BI into the autophagosome-associated/lipidated form LC3BII) and of the autophagy receptor p62 (SQSTM1/p62) [20]. Alongside, PC1 protein level was also assessed. Interestingly, PC1 decreased in cells treated with Torin 1 and increased in cells treated with the autophagic inhibitor BafA1, if compared to untreated cells (Figs. 2B–E and S2A–G). Immunofluorescence analyses of tendon cells treated with BafA1 further confirmed the intracellular accumulation of PC1 (Fig. 2F, G) and highlighted that a fraction of PC1 colocalized with LC3B (Fig. 2H–I and S3A) suggesting that, in tendon cells, PC1 is a substrate of autophagy.

We then speculated that the biological relevance of these observations was that autophagy could be required to eliminate misfolded PC1. To test this hypothesis we took advantage of the fact that when PC1 fails to fold properly into a triple helix, it accumulates as detergent-insoluble aggregates [21–23]. Thus, to evaluate whether the BafA1-induced accumulation of PC1 was due to the formation of aggregates, we performed a centrifugal fractionation assay followed by western blotting. Although most of the PC1 was in the soluble fraction, a significant amount of the protein was also detected in the insoluble fraction (Fig. S4A–C). To give strength to this evidence, we knocked down the *autophagy-related gene 7* (ATG7), which is involved in AVs biogenesis and, next, performed immunofluorescence and western blot analyses of PC1. Both these approaches showed that, when autophagy is inhibited, PC1 accumulates in tendon cells (Fig. 2J), this phenomenon being even more evident in the insoluble fraction (Fig. 2K–M). This result was coherent with the observed impairment of autophagy in cells downregulating Atg7 (siAtg7) (Fig. 2N–P) that showed a higher accumulation of PC1 after BafA1 treatment than control (siSCR) cells (Fig. 2Q, R). These data indicated that autophagy plays a role in preventing PC1 intracellular accumulation, reasonably by degrading the fraction of procollagen molecules that undergo misfolding and aggregation.

### Autophagy prevents intracellular accumulation of procollagen I at the ER via selective ER-phagy

**Fig. 3:**
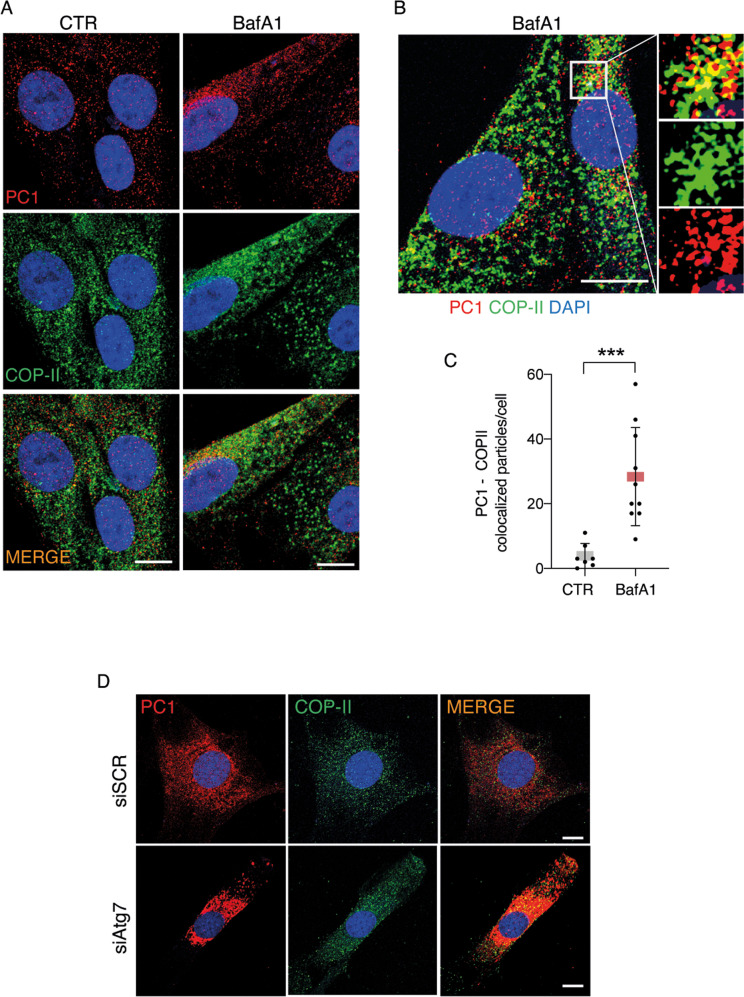
Inhibition of autophagy results in accumulation of PC1 in the ER exit sites in primary human tendon cells. **A** Human tendon cells control and treated with BafA1 for 16 h were immunolabeled for PC1 (red), COPII (green), and nuclei stained with DAPI (blue) and analyzed by confocal microscopy. Scale bar = 10 μm. **B** Cells treated with BafA1 for 16 h were immunolabeled for PC1 (red), COP-II (green), and nuclei stained with DAPI (blue) and analyzed by confocal microscopy. The insets show higher magnification and single color channels of the boxed area. Scale bars = 10 μm. **C** Quantification of PC1-COP-II colocalized particles per cell. ***p < 0.001, unpaired *t*-test. **D** Confocal microscopy images of control and siAtg7-treated cells immunolabeled for PC1 (red), COP-II (green), nuclei stained with DAPI (blue). Scale bar = 10 μm.

**Fig. 4:**
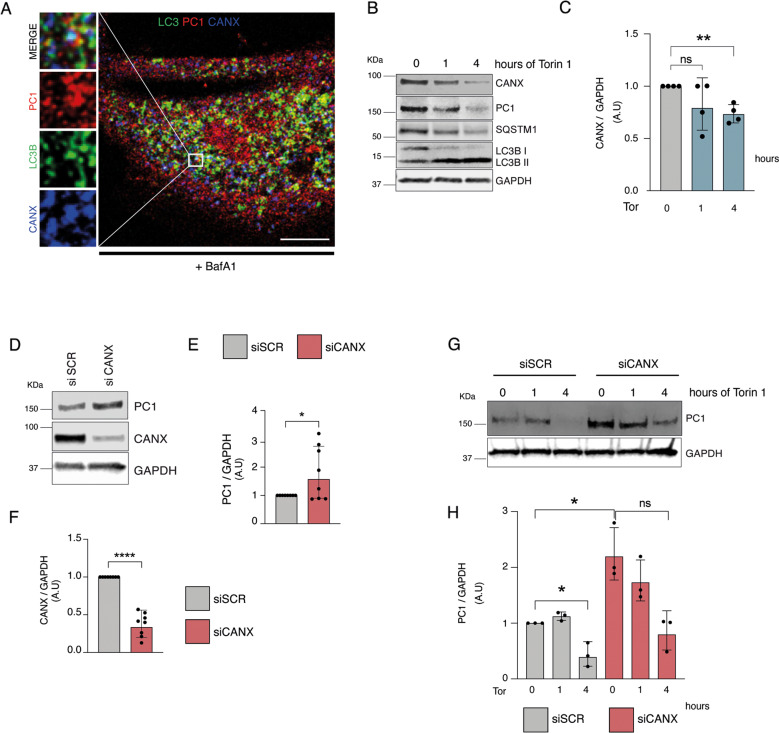
Calnexin is involved in the degradation of PC1 in primary human tendon cells. **A** Human tendon cells control and treated with BafA1 for 16 h were immunolabeled for PC1 (red), CANX (blue), and LC3B (green) and analyzed by confocal microscopy. The insets show higher magnification and single color channels of the boxed area. Scale bars = 10 μm. **B** Western blot analysis of CANX, PC1, SQSTM1/p62, and LC3B in control and Torin 1 (Tor)-treated cells for 1 and 4 h. GAPDH was used as a loading control. **C** Quantification of CANX western blot data. Data are representative of four independent experiments made with cells from four different human donors. **p < 0.01, ***p < 0.001, unpaired *t*-test. **D** Western blot analysis of PC1 and CANX, in siSCR and siCANX cells. GAPDH was used as a loading control. Quantification of the normalized PC1 (**E**) and CANX (**F**) proteins levels. Data are representative of eight independent experiments made with cells from three different human donors. *p < 0.05, ****p < 0.0001, unpaired *t*-test. **G** Western blot analysis of PC1 and SQSTM1/p62, siSCR, and siCANX cells treated with Torin 1 for 1 and 4 h. GAPDH was used as a loading control. **H** Quantification of the normalized PC1 protein levels. Data are representative of three independent experiments made with cells from three different human donors. *p < 0.05, unpaired *t*-test.

**Fig. 5:**
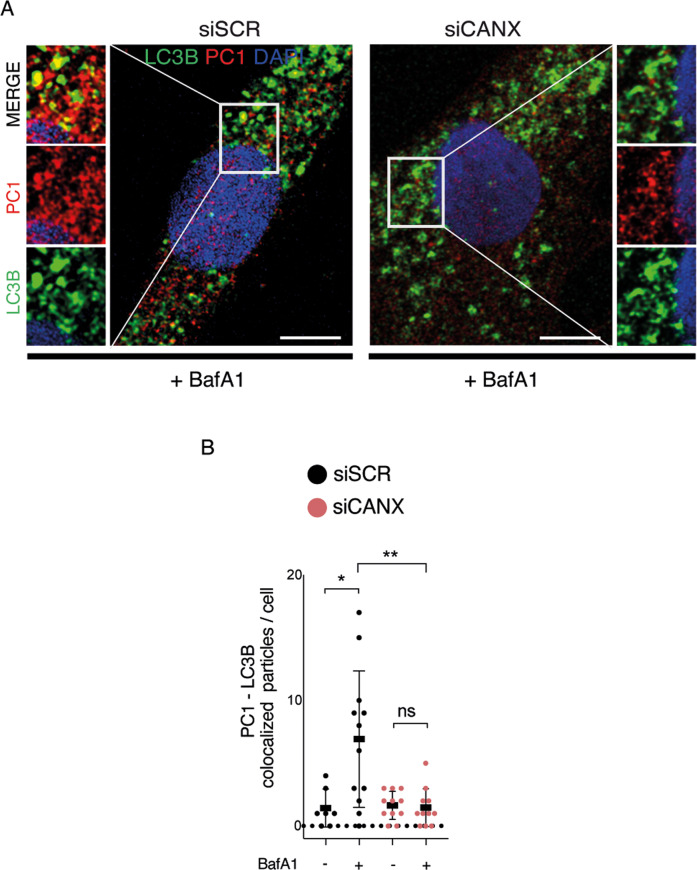
Calnexin is required for autophagy of PC1 in primary human tendon cells. **A** Control and siCANX-treated human tendon cells treated with BafA1 for 16 h were immunolabeled for PC1 (red), LC3B (green), nuclei stained with DAPI (blue) and analyzed by confocal microscopy. The insets show higher magnification and single color channels of the boxed area. Scale bars = 10 μm. **B** Quantification of PC1-LC3B colocalized particles per cell. *p < 0.05, **p < 0.01, unpaired *t*-test.

ER-phagy is initiated at two different subdomains of the ER, i.e., at the ER exit sites [16, 23] and at a modified form of ERES, ER-phagy sites [24]. Both subdomains are decorated with the coat protein complex II (COPII) and arise through the activities of COPII [16, 24]. To understand if PC1 becomes an autophagy substrate while it is trafficked in the ER, we first evaluated the intracellular localization of PC1. Immunofluorescence analysis showed a nice colocalization of PC1 and COPII in tendon cells treated with BafA1 (Fig. 3A–C) and in siAtg7-treated tendon cells (Figs. 3D and S3B, C). Next, we investigated the involvement of selective ER-phagy mediated by RETREG1/FAM134B-calnexin complex. Immunofluorescence analysis confirmed colocalization between PC1, LC3B, and the ER chaperone calnexin (CANX) (Figs. 4A and S3D–F), showing that PC1 and CANX are found together in the AVs and that PC1 is sequestered within the AVs when they are still retained in the ER. Western blot analysis of lysates from cells treated with Torin1 revealed that CANX itself is a substrate of autophagy together with PC1 (Fig. 4B and C), and CANX downregulation by siRNAs (siCANX) caused an increase in PC1 protein level (Fig. 4D–F), arguing for a role of CANX in mediating PC1 degradation via autophagy. Consistently, the induction of autophagy by Torin 1 treatment did not significantly affect PC1 protein level in siCANX cells (Fig. 4G-H). Moreover, CANX silencing resulted in less PC1 recruitment by AVs upon BafA1-mediated autophagy inhibition (Figs. 5A, B and S3G), giving further support to the hypothesis that CANX is required for autophagy-mediated degradation of PC1 in tendon cells.

### Activation of autophagy by inhibition of mTor pathway influences the thickness and morphology of 3D tissue-engineered tendons

**Fig. 6:**
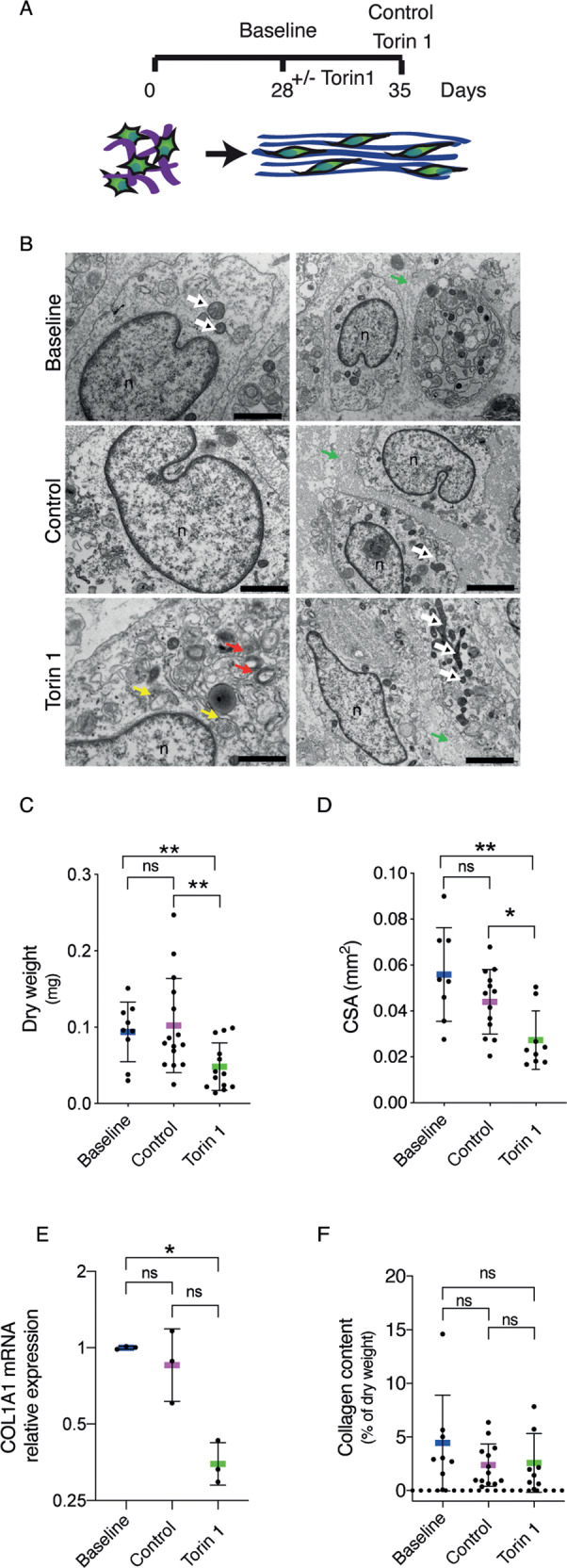
Pharmacological inhibition of mTOR by Torin 1 has a negative effect on thickness of 3D tissue-engineered tendons. **A** Experimental design of treatment of 3D tissue-engineered tendons from day 28 after cell seeding (Baseline), or treated with Torin 1 until day 35 (Torin1) or left as control until harvesting until day 35 (Control). **B** Representative transverse images showing cell morphology of 3D tissue-engineered tendons analyzed by TEM. Nuclei (n), mitochondria (white arrows), fibropositors (green arrows), autophagosomes (yellow arrows), and autophagolysosomes (red arrows) are indicated. Left scale bars = 1 μm; right scale bars = 2 μm. **C** Dry weight and cross-sectional area (CSA) (**D**), were determined in baseline, control and Torin1 samples. *n* = 3 tissue donors, with a minimum of 8 tissue-engineered tendons per cell preparation for each treatment for each parameter measured, *p < 0.05, **p < 0.01, unpaired t-test. **E** *COL1A1* gene expression in baseline, control, and Torin1 samples. Data are representative of three independent experiments made with cells from three different human donors. *p < 0.05, paired *t*-test, Bonferroni corrected. **F** Collagen content, normalized on the dry weight, was also determined. *n* = 3 tissue donors, with a minimum of 8 tissue-engineered tendons per cell preparation for each treatment for each parameter measured, *p < 0.05, **p < 0.01, unpaired *t*-test.

**Fig. 7:**
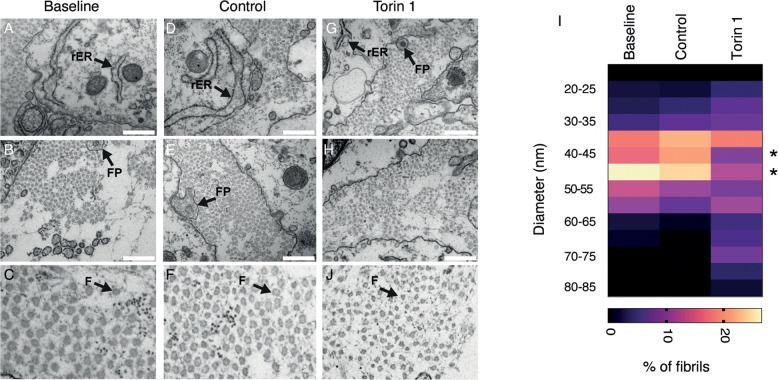
Pharmacological inhibition of mTOR by Torin 1 impairs collagen fibrils morphology. **A–H** Representative transverse images of 3D tissue-engineered tendons analyzed by TEM. Baseline and control samples showed fibrils with similar fibril diameter and regular shapes (**A–F**). Torin 1 samples, in contrast, showed irregular fibrils (**G, H**). rER rough endoplasmic reticulum, FP fibripositors, F collagen fibril. Scale bar = 500 nm. *n* = 3 tissue donors in each experimental group. **I** Heat map plot of the distribution of fibril diameters measured in TEM images of baseline, control and Torin1 3D tissue-engineered tendons. Measurements from at least 800 fibrils per sample measured. *n* = 3 tissue donors in each experimental group. *p < 0.05, unpaired *t*-test of Torin 1 vs control samples.

To evaluate whether autophagy is involved in tendon tissue homeostasis, we used tendon-like tissue made from human tendon cells in vitro. This three-dimensional (3D) tissue-engineered human tendon is an innovative experimental model for studying fibril formation [25]. The fibrils of these tissue-engineered tendons are synthesized and assembled by tendon cells and arranged in parallel bundles (such as in tendons and ligaments) with features recapitulating those of tendons in vivo [26]. The 3D tissue-engineered tendons were evaluated by different parameters (i.e., dry weight, cross-sectional area, collagen gene expression and protein content, collagen fibrils diameter) after 28 days from seeding (baseline) compared with those assessed after one additional week in culture (35 days in total after seeding), in which tendon cells were grown in the presence or absence of Torin 1 (see scheme in Fig. 6A). Transmission electron micrographs showed healthy cells and a massive formation of vesicles resembling autophagosome/autolysosome-like bodies in Torin1-treated 3D tissue-engineered tendons (Fig. 6B). The induction of autophagy was also confirmed by Western blot showing an increase in LC3BII:LC3BI ratio along with a reduction of SQSTM1/p62 protein levels (Fig. S5A and B). Conversely, LC3B and SQSTM1/p62 mRNA expression showed no statistically significant differences (Fig. S5C). Dry weight and cross-sectional area were reduced in 3D tissue-engineered tendons treated with Torin1 (Fig. 6C and D). Among the collagen genes expressed in tendons, only *COL1A1* gene displayed reduced expression in 3D tissue-engineered tendons treated with Torin 1 (Figs. 6E and S5D). The overall collagen content was comparable in all 3D tissue-engineered tendon samples (Fig. 6F). The DNA content did not show any difference (Fig. S5E). There were no statistically significant differences in any of the above parameters between the baseline and the control 3D tissue-engineered tendons (Fig. 6C–F), and, in general, the expression of other genes contributing to tendon formation (e.g., those involved in ECM production) did not seem to be significantly affected by Torin 1 treatment (Fig. S5F, G). Electron microscopy analyses were carried out to analyze tissue morphology. Transverse sections showed similar regular fibrils with circular outlines in the extracellular space, and properly functioning cells with intact membranes and rough ER (Fig. 7A, D and G). At higher magnification, the collagen fibrils size appeared comparable in baseline and control samples (Fig. 7C, F). The presence of specialized cell protrusions containing newly synthesized collagen fibrils called fibripositors indicates a newly synthesized matrix (Fig. 7B, E and G). Fibripositors were also present in Torin1 samples (Fig. 7J). However, Torin1 tissue-engineered tendons showed irregular collagen fibrils (Fig. 7H and J). In support of this observation, the diameters of the fibrils in 3D tissue-engineered tendons upon Torin1 treatment lost the characteristic distribution of fibrils in the baseline and control tissue-engineered tendons, with a significant decrease in the middle size fibers (40–50 nm), in favor of the smaller and larger fibrils (Fig. 7I).

### Activation of autophagy by inhibition of mTor pathway influences tendon material properties

**Fig. 8:**
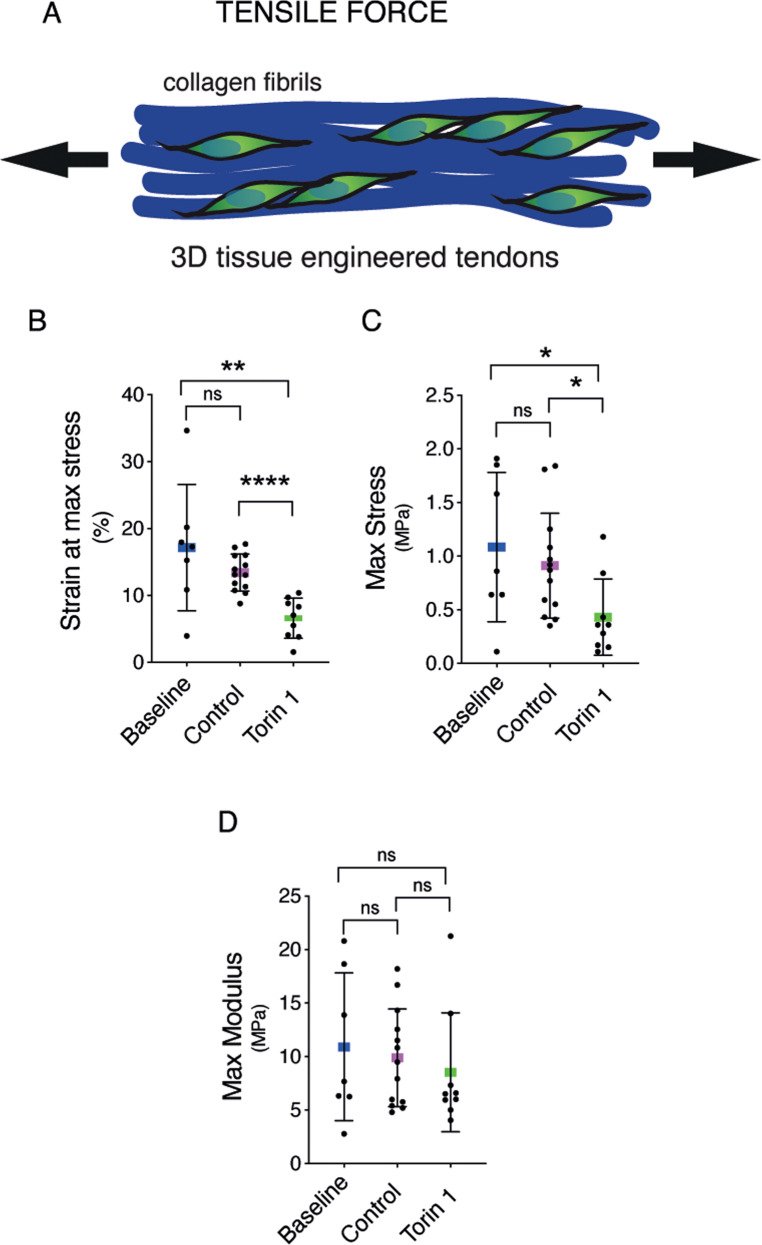
Pharmacological inhibition of mTOR by Torin 1 affects matrix quality. **A** Graphical representation of the tensile force applied to the tissue-engineered tendons. Maximum stress (**B**), strain at maximum stress (**C**), and maximum modulus (**D**). *n* = 3 tissue donors, with a minimum of 7 tissue-engineered tendons per cell preparation for each treatment for each parameter measured, *p < 0.05, **p < 0.01, ****p < 0.0001, unpaired *t*-test.

To assess whether modulation of autophagy alters mechanical properties, we treated 3D tissue-engineered tendons with Torin 1 and measured mechanical strength under tensile force (Fig. 8A). The maximum stress and maximum strain were both significantly reduced in Torin1-treated engineered tendons if compared with both the baseline and the control counterparts (Fig. 8B and C). Conversely, no differences were observed for the elastic modulus (Fig. 8D), with baseline and control samples showing similar values in all the measurements performed (Fig. 8B–D). These results represent a proof of the concept that a constant autophagy flux of tendon cells is implicated in defining and maintaining the correct structure of tendon, likely by sustaining the intracellular degradation of misfolded PC1.

## Discussion

In this study, we have provided an essential and, to our knowledge, previously undocumented role of autophagy in the regulation of PC1 synthesis and homeostasis in human tendon cells. The presence of autophagosome-containing PC1 in healthy tendons tissue, together with the accumulation of PC1 in the insoluble fraction when autophagy is impaired, supports the hypothesis that autophagy preferentially degrades misfolded PC1 molecules to prevent their accumulation in the ER, as it has been recently discovered by Forrester et al. in mouse embryonal fibroblasts and osteosarcomas cells [18]. As a matter of fact, we found out that any alteration of autophagy flux—ectopically induced in tendon cells by both inhibiting (with BafA1 and siAtg7) and enhancing autophagy flux (with Torin1)—affected the correct synthesis of PC1, tendon homeostasis, and properties. In particular, we observed that the inhibition of autophagy results in the accumulation of PC1 aggregates in the ER subdomains specifically targeted by CANX-mediated ER-phagy. CANX supports the folding of mono-glycosylated glycoproteins in the ER, creating transient complexes with unfolded ER proteins until they become folded or degraded [27]. However, a new pathway has recently been discovered by which the ER-resident autophagy receptor RETREG1/FAM134B interacts with both CANX and LC3B, originating a specific ER-phagy complex. This complex is capable of a specific mechanism for PCs removal, which connects non-native proteins within the ER lumen to the cytosolic autophagy machinery [18]. In tendon cells, we have demonstrated the formation of the CANX-PC1-LC3B complex, indicating that PC1 becomes an autophagy substrate while it is still retained in the ER.

Finally, we have also provided evidence that pharmacological activation of autophagy by Torin 1 affects extracellular collagen fibrils and mechanical properties of 3D tissue-engineered tendons. Torin 1 is a potent and selective inhibitor of the mechanistic target of rapamycin kinase (mTOR) kinase [28, 29], that coordinates and promotes cell growth and proliferation. mTOR pathway negatively controls autophagy, and Torin 1, as inhibition of mTOR, mimics cellular starvation and induces autophagy [30, 31]. In yeast, which is the pioneer biological model for autophagy research, it has been found that Tor inhibition causes the upregulation of one of the two so far identified ER-phagy receptors, Atg40 [32]. Atg40 has a domain structure similar to the mammalian RETREG1/FAM134B, and in mammals—as well as in yeast—mTOR inhibition stimulates the delivery of ER to the lysosome [24].

In tendon, mTOR signaling is upregulated during in vitro tenogenic differentiation of mesenchymal stem cells, whereas tendon-specific ablation of mTOR in mice results in hypoplasia of tendons, along with impairment of biomechanical properties [33]. Notably, among other connective tissues, cartilage has been extensively reported to be regulated by autophagy [34, 35]. Cartilage, similar to the tendon, is composed of a large amount of collagenous extracellular matrix, and mTOR inhibition is associated with less severity of cartilage degeneration [36, 37]. It has been demonstrated that autophagy protects cartilage integrity by the removal of damaged mitochondria and, in turn, counteracts oxidative stress [38]. However, another potential pathogenic mechanism in cartilage degeneration is the increased chondrocyte ER stress [39], but the role of selective autophagy of the ER has not been investigated so far.

In this study, we provide new in vitro and in vivo evidence that autophagy is also involved in tendon physiology. In particular, we propose that autophagy acts as a quality control mechanism underlying PC1 correct maturation, thereby contributing to proper ECM synthesis and mechanical properties of tendon tissue. As the ability of the tendon to remodel the ECM has been suggested to be a risk factor for tendon pathologies [7–11], this pathway might represent a crucial process involved in the development of tendon injuries. However, if autophagy is beneficial or detrimental for tendons still needs to be demonstrated. The use of tendon-specific conditional *Atg5* or *Atg7* KO mice could in the future help better define this issue. It is, indeed, worth recalling that autophagy is a Janus-faced process, with defects or over-induction both being deleterious for cells. This is probably the case also in tendon homeostasis and correct collagen maturation, and we believe that autophagy may bridge the lack of knowledge behind the understanding of tendinopathy.

## Materials and methods

### Primary human tendon cells

Tendon fibroblasts were isolated from human *gracilis* tendons from patients that underwent reconstructive anterior cruciate ligament surgery. The tissue was minced into small pieces and digested overnight in DMEM/F-12 (21041025, Gibco) supplemented with 0.1% collagenase type 2 (LS004176, Worthington) and 20% fetal bovine serum (FBS) (S1810-100, BioWest). Cells were cultured in complete DMEM/F-12 (20% FBS and 100 U/ml penicillin and 100 μg/ml streptomycin) and used for experiments between passages 2 and 8. Cells from ten different donors were used for experiments. Cells were checked regularly by PCR for the presence of mycoplasma and all cells used were negative.

### Transfections and treatments

Transient knocking down of ATG7 and CANX was performed by transfecting cells with endoribonuclease-prepared siRNA pool (siAtg7, EHU092581, Sigma–Aldrich), or Silence Select Pre-Designed siRNA (siCANX, s2377, Thermo Fisher), while controls were transfected with a scrambled siRNA duplex (siScr, SIC001, Sigma–Aldrich). siRNAs were transfected using Lipofectamine RNAiMAX Transfection Reagent (13778075, Thermo Fisher Scientific), according to the manufacturer’s instructions. Cells were harvested 72 h after transfection. Bafilomycin A1 (B-1080, LC-lab) was used at a final concentration of 200 nM for 4 and 16 h, as previously reported [18]. Torin 1 (T-7887, LC-lab) was used at a final concentration of 1 μM for 1, 2, 4, and 24 h, as previously described [40].

### Immunofluorescence (IF) and microscopy

Mouse tendon tissues: Mouse experiments were carried out in accordance with the European Community guidelines and with the approval of relevant National and local ethical committees. C57BL/6 wild-type was purchased from Charles River. Mice were housed in an environmentally controlled room (23 °C, 12 h light–dark cycle) and provided with food and water ad libitum. On cervical dislocation, Achilles tendons of five 8-weeks-old mice were dissected, embedded in Tissue-Tek O.C.T. (4583, Sakura), flash-frozen in liquid nitrogen-cooled isopentane (TCM0167, VWR), and stored at −80 °C until analysis, as previously described [41]. The longitudinal sections (8 μm thick) were obtained with a Thermo Fisher cryostat (−20 °C) and used for immunostaining. No exclusion criteria, randomization, and blinding were applied.

Human tendon tissues: *gracilis* tendons from seven patients that underwent ACL surgery were collected and embedded in Tissue-Tek O.C.T. and flash-frozen in liquid nitrogen-cooled isopentane, as described above. Mean age of 30 ± 9.7 SD, 4 males, 3 females, of normal BMI (18.5–24.9).

Cells: cells were grown on plastic coverslips. For autophagy analyses (LC3B staining), cells were fixed and permeabilized in ice-cold MeOH for 10 min at −20 °C. For localization in the ER (COP-II staining), cells were fixed in 4% formaldehyde for 10 min at room temperature (RT) and permeabilized in PBS plus 0.2% triton X-100 for 2 min at RT. After incubation with primary and secondary antibodies (Alexa Fluor 488, 568, or 680, Life Technologies), the slides were mounted in ProLong Gold Antifade Mountant with DAPI (P36935, Life Technologies). Immunofluorescence analyses were acquired on 0.3 μm thick slices using the laser scanning confocal microscopes (LSM700, Carl Zeiss A/S), equipped with a 63 × 1.4 numerical aperture oil objective.

### IF quantification

Fluorescence images were adjusted for brightness, contrast, and color balance by using Fiji analysis software. Confocal microscopy images were deconvoluted using the software Huygens Professional (Scientific Volume Imaging). The number of particles in which PC1 colocalizes with LC3B was calculated by Fiji analysis software using the open-source plugin ComDet v. 0.3.7. on at least eight different cells per experimental condition. For both PC1 and LC3B fluorescence channels, the parameters utilized were: Particle size ≥6 px; intensity threshold = 3. For COPII fluorescence channels, the parameters utilized were: Particle size ≥8 px; intensity threshold = 4. The colocalization was considered positive if the maximum distance between the center of 2 particles was ≤5 px. Pearson’s and Manders’ coefficients were calculated by Fiji ImageJ using the plugin JaCoP v2.0 [42] with Costes’ thresholding method [43].

### Histology

Tendon sections were stained in hematoxylin & eosin and picrosirius red following standard procedures.

### Immunoblotting (IB)

Cells were washed twice with PBS and then scraped in RIPA lysis buffer (R0278, Sigma) containing protease (5892791001, Roche) and phosphatase inhibitors (4906845001, Roche) at 4 °C for 30 min and centrifuged for 30 min at 22,300 × *g* at 4 °C. Protein extracts were quantified using the BCA protein assay (23225, Thermo Fisher), and denatured in 1× NuPAGE LDS Sample Buffer (NP0008, Life technologies) containing the 1x NuPAGE Sample Reducing Agent (NP0009, Life technologies). Samples were then boiled at 100 °C for 5 min. Proteins were separated on polyacrylamide gradient gels (5671084, Bio-Rad) and blotted onto polyvinylidene difluoride (PVDF) membranes (Bio-Rad). For the centrifugal fractionation assay, after the centrifugation the supernatant fractions (soluble fraction) were collected. The pellet fractions (insoluble fraction) were washed three times in RIPA buffer, then, the dry pellet was resuspended in 1× NuPAGE LDS Sample Buffer containing 1× NuPAGE Sample Reducing Agent.

### Antibodies

Primary antibodies were as follows: Col-I (Abcam, ab34710, IB 1:1000), LC3B (Cell Signaling Technology, 2775, IB: 1:1000), Atg7 (Cell Signaling Technology, 2631, IB: 1:1000), SQSTM1/p62 (MBL, PM045, IB: 1:1000), GAPDH (Thermo Fisher, AM4300, IB: 1:10000), CANX (Enzo Life Sciences, ADI-SPA-860, IB 1:1000), LC3B (Cell Signaling Technology, 2775, IF: 1:100), LC3B (Nanotools, 5F10, IF 1:250, only used in co-IF with CANX ab), PC1 (Abcam, ab64409, IF 1:100), COPII (Invitrogen Life Technologies, PA1-069A, IF 1:00), LAMP1 (Abcam, ab24170, IF 1:150), Atg12 (Cell Signaling Technology, 2010, IF 1:100), Phospho-mTOR Ser2448 (Cell Signaling Technology, 2971, IB: 1:1000), mTOR (Cell Signaling Technology, 2983, IB: 1:1000), Phospho-p70 S6 Kinase Thr389 (Cell Signaling Technology, 9205, IB: 1:1000).

### 3D tissue-engineered tendons

3D tissue-engineered tendons from human tendon fibroblasts were assembled as previously described [26]. Human tendon fibroblasts were suspended in a mix of fibrinogen (F3879, Sigma), aprotinin (A6103, Sigma), and thrombin (T4393, Sigma) to a final concentration of 0.2 million cells per well and rapidly spread over the complete surface of the Sylgard (SYLG184, Farnell) coated wells. The gels were incubated in a constructed medium (DMEM/F-12, 10% FBS, 0.2 mM L-ascorbic acid 2-phosphate, A8960, Sigma, 0.05 mM L-proline, P5607, Sigma), which was replaced every other day. Approximately 2 weeks after seeding the cells had contracted the gel to a 10 mm long narrow linear structure between the sutures and on day 28 the 3D tissue-engineered tendons were completely formed. After that, 3D tissue-engineered tendons were supplemented with 1 μM Torin 1 or left as control until harvesting (day 35).

### Dry weight, collagen, and DNA content assays

After mechanical testing, tendon constructs were snap-frozen in liquid nitrogen and stored at −80 °C until further use. Samples were freeze-dried and weighed at constant humidity using an ultra-microbalance scale (Mettler-Toledo GmBH, Gieben, Germany). Hydroxyproline and DNA content was measured as previously described [44].

### Mechanical testing

Tensile testing of the engineered tendons was performed at baseline (28 days after cell seeding), control, and Torin 1-treated samples (35 days after cell seeding). The testing was performed in a PC-driven micromechanical rig with a liquid chamber (20 N load-cell, sampling rate 10 Hz; Deben, Suffolk, UK). A stereoscopic microscope (SMZ1000, Nikon, Tokyo, Japan) equipped with a digital camera (SC50, Olympus) was used for imaging during the test to verify the length and monitor the rupture site of the tissue-engineered tendons. The engineered tendons were attached by their silk suture loops onto specimen plates containing hooks and tested in a cell culture medium-bath after a short adaptation period. The samples were stretched at 2 mm/min until the onset of force and after a 15 s relaxation period, the constructs were further stretched at 2 mm/min until failure. The diameter and mounting length were measured on microscopy images at the onset before the stretch to failure. The diameter was measured in four different places and an average cross-sectional area was calculated assuming a circular cross-section.

### Transmission electron microscopy

Tendon constructs were fixed with 2.5% glutaraldehyde for at least 24 h and prepared at the University of Copenhagen, Core Facility for Integrated Microscopy. Images were acquired on 70 nm thick axial sections with a CM 100 BioTWIN electron microscope. Fibril diameters were measured with the aid of a custom MATLAB script (MATLAB 2017b, The MathWorks Inc.) that automatically detects and measures fibril diameters using a form of circular Hough transform [45]. The results were subsequently curated manually to remove or add any incorrect or missing fibril measurements.

### Real-time quantitative PCR

RNA was isolated from 3D tissue-engineered tendons and mRNA expression was measured as described previously [46]. Real-time RT-qPCR was performed using SYBR-Green Technology with the primers specified in Supplementary Table 1. To define a suitable reference gene, five different housekeeping genes were measured (RPLP0, GAPDH, b2M, S26, and CycA/PPIA). The online tool RefFinder (https://pubmed.ncbi.nlm.nih.gov/22290409) was used for defining the most stable among those. CycA/PPIA came out as the best-ranking mRNA for normalization in this study and was therefore used for the normalization of the target mRNAs.

### Statistical analyses

Statistical analyses were performed with Prism software (GraphPad Software). Unless otherwise stated, error bars in bar plots were represented means ± SD, and the analyses were performed using the two-tailed unpaired *t*-test. For gene expression analyses paired *t*-tests were performed on log-transformed values and *p*-values were Bonferroni corrected (multiplied by 3) to adjust for multiple comparisons and error bars in bar plots represented as geometrical means ± geometrical SD. For all figures statistics are defined as: ns *p* > 0.05, *p < 0.05, **p < 0.01, ***p < 0.001, ****p < 0.0001. No statistical method was used to predeterminate the sample size. Untreated cells, with drug-vehicle, or cells transfected with siSCRAMBLE siRNA were included as appropriate controls for each specific experiment.

## Supplementary information

agreement on author’s list

Supplementaries figure legends

Figure S1

Figure S2

Figure S3

Figure S4

Figure S5

Supplementary Table 1

Original Western Blots

Reproducibility checklist

## Data Availability

All data needed to evaluate the conclusions in the paper are present in the paper. Additional data related to this paper may be requested from the corresponding author.
