# Supplementary material for: Autophagy guards tendon homeostasis
Source: Cell Death Dis. 2022 Apr 23;13(4):402. doi: 10.1038/s41419-022-04824-7 (PMC9035152; doi:10.1038/s41419-022-04824-7)
Supplement: Supplementary file 1 — agreement on author’s list [file 41419_2022_4824_MOESM1_ESM.pdf]

From: **Michael Kjær** michaelkjaer@sund.ku.dk
Subject: Re: CDDis IMPORTANT agreement
Date: 31 March 2022 at 13:22
To: Costanza Montagna costanzamontagna@gmail.com, Rene Svensson svensson.nano@gmail.com, Monika Lucia Bayer Monika.Lucia.Bayer@regionh.dk, Monika Bayer mlbayer82@gmail.com, Chloé Yeung chloe.yeung@gmail.com

MK

I hereby confirm that i agree in the including Emiliano Maiani as an author of the new version of the paper by corresponding author Costanza Montagna, where i myself is a co-author. Emiliano Maiani has contributed significantly contributed to the additional experiment requested by the reviewers. I hereby agree with the change of authorship list to: Costanza Montagna, Rene Svensson, Monika Bayer, Salvatore Rizza, Emiliano Maiani, Chloé Yeung, Giuseppe Filomeni, Michael Kjaer.
kind regards
Michael Kjaer

From: Costanza Montagna <costanzamontagna@gmail.com>
Sent: Thursday, March 31, 2022 12:17:15 PM
To: Michael Kjær; Rene Svensson; Monika Lucia Bayer; Monika Bayer; Chloé Yeung
Subject: CDDis IMPORTANT agreement

Dear co-authors,

I need a formal agreement from all of you because I have included in the new version a new author, Emiliano Maiani, who helped me in the further analysis of immunofluorescence, as requested by the reviewers.
If you can reply to this mail confirming that you agree with the change, I will proceed with the final submission.

The previous author list was:

Costanza Montagna, Rene Svensson, Monika Bayer, Salvatore Rizza, Chloè Yeung, Giuseppe Filomeni, Michael Kjaer.

Now:

Costanza Montagna, Rene Svensson, Monika Bayer, Salvatore Rizza, Emiliano Maiani, Chloé Yeung, Giuseppe Filomeni, Michael Kjaer.

Thanks

Warm regards

Costanza

From: **René Svensson** svensson.nano@gmail.com
Subject: Re: CDDis IMPORTANT agreement
Date: 31 March 2022 at 14:08
To: Monika Lucia Bayer Monika.Lucia.Bayer@regionh.dk
Cc: Costanza Montagna costanzamontagna@gmail.com, michaelkjaer@sund.ku.dk, Monika Bayer mlbayer82@gmail.com, Chloé Yeung chloe.yeung@gmail.com

RS

Dear Costanza,

I, Rene Svensson, hereby confirm including Emiliano Maiani as an author of the new version of the paper "Autophagy guards tendon homeostasis" by corresponding author Costanza Montagna. Emiliano Maiani has significantly contributed to the additional experiment requested by the reviewers. I hereby agree with the change of authorship list to: Costanza Montagna, Rene Svensson, Monika Bayer, Salvatore Rizza, Emiliano Maiani, Chloé Yeung, Giuseppe Filomeni, Michael Kjaer.

Best regards,
Rene Svensson

Virus-free. www.avg.com

On Thu, Mar 31, 2022 at 1:55 PM Monika Lucia Bayer <Monika.Lucia.Bayer@regionh.dk> wrote:

> I hereby confirm including Emiliano Maiani as an author of the new version of the paper by corresponding author Costanza Montagna, where I, Monika Bayer, am a co-author. Emiliano Maiani has significantly contributed to the additional experiment requested by the reviewers. I hereby agree with the change of authorship list to: Costanza Montagna, Rene Svensson, Monika Bayer, Salvatore Rizza, Emiliano Maiani, Chloé Yeung, Giuseppe Filomeni, Michael Kjaer.
>
> Sincerely yours
> /Monika Bayer
>
> -----Oprindelig meddelelse-----
> Fra: Costanza Montagna <costanzamontagna@gmail.com>
> Sendt: 31. marts 2022 12:17
> Til: michaelkjaer@sund.ku.dk; svensson.nano@gmail.com; Monika Lucia Bayer <Monika.Lucia.Bayer@regionh.dk>; Monika Bayer <mlbayer82@gmail.com>; Chloé Yeung <chloe.yeung@gmail.com>
> Emne: CDDis IMPORTANT agreement
>
> Dear co-authors,
>
> I need a formal agreement from all of you because I have included in the new version a new author, Emiliano Maiani, who helped me in the further analysis of immunofluorescence, as requested by the reviewers.
> If you can reply to this mail confirming that you agree with the change, I will proceed with the final submission.
>
> The previous author list was:
>
> Costanza Montagna, Rene Svensson, Monika Bayer, Salvatore Rizza, Chloè Yeung, Giuseppe Filomeni, Michael Kjaer.
>
> Now:
>
> Costanza Montagna, Rene Svensson, Monika Bayer, Salvatore Rizza, Emiliano Maiani, Chloé Yeung, Giuseppe Filomeni, Michael Kjaer.
>
> Thanks
>
> Warm regards
>
> Costanza
>
> ________________________________
>
> Region Hovedstaden anvender de personoplysninger, du giver os i forbindelse med din henvendelse. Du kan læse mere om formålet med anvendelsen samt dine rettigheder på vores hjemmeside: www.regionh.dk/persondatapolitik

--
Rene B. Svensson
Postdoc, Ph.D. Health Science, M.Sc. Nanotechnology

Institute of Sports Medicine Copenhagen
Bispebjerg Hospital
Nielsine Nielsens Vej 11, Building 8, 1st floor
2400 Copenhagen NV

From: **Monika Lucia Bayer** Monika.Lucia.Bayer@regionh.dk
Subject: SV: CDDis IMPORTANT agreement
Date: 31 March 2022 at 13:55
To: Costanza Montagna costanzamontagna@gmail.com, michaelkjaer@sund.ku.dk, svensson.nano@gmail.com, Monika Bayer mlbayer82@gmail.com, Chloé Yeung chloe.yeung@gmail.com

MB

I hereby confirm including Emiliano Maiani as an author of the new version of the paper by corresponding author Costanza Montagna, where I, Monika Bayer, am a co-author. Emiliano Maiani has significantly contributed to the additional experiment requested by the reviewers. I hereby agree with the change of authorship list to: Costanza Montagna, Rene Svensson, Monika Bayer, Salvatore Rizza, Emiliano Maiani, Chloé Yeung, Giuseppe Filomeni, Michael Kjaer.

Sincerely yours
/Monika Bayer

-----Oprindelig meddelelse-----
Fra: Costanza Montagna <costanzamontagna@gmail.com>
Sendt: 31. marts 2022 12:17
Til: michaelkjaer@sund.ku.dk; svensson.nano@gmail.com; Monika Lucia Bayer <Monika.Lucia.Bayer@regionh.dk>; Monika Bayer <mlbayer82@gmail.com>; Chloé Yeung <chloe.yeung@gmail.com>
Emne: CDDis IMPORTANT agreement

Dear co-authors,

I need a formal agreement from all of you because I have included in the new version a new author, Emiliano Maiani, who helped me in the further analysis of immunofluorescence, as requested by the reviewers.
If you can reply to this mail confirming that you agree with the change, I will proceed with the final submission.

The previous author list was:

Costanza Montagna, Rene Svensson, Monika Bayer, Salvatore Rizza, Chloè Yeung, Giuseppe Filomeni, Michael Kjaer.

Now:

Costanza Montagna, Rene Svensson, Monika Bayer, Salvatore Rizza, Emiliano Maiani, Chloé Yeung, Giuseppe Filomeni, Michael Kjaer.

Thanks

Warm regards

Costanza

____

Region Hovedstaden anvender de personoplysninger, du giver os i forbindelse med din henvendelse. Du kan læse mere om formålet med anvendelsen samt dine rettigheder på vores hjemmeside: www.regionh.dk/persondatapolitik

From: **salvatore rizza** salvatoreri@gmail.com
Subject: Authors' list Change
Date: 31 March 2022 at 13:15
To: Costanza Montagna costanzamontagna@gmail.com

SR

Dear Costanza,
I agree with the change in authors' list.

The previous author list was:

Costanza Montagna, Rene Svensson, Monika Bayer, Salvatore Rizza, Chloè Yeung, Giuseppe Filomeni, Michael Kjaer.

Now:

Costanza Montagna, Rene Svensson, Monika Bayer, Salvatore Rizza, Emiliano Maiani, Chloé Yeung, Giuseppe Filomeni, Michael Kjaer.

Best regards,

Salvatore Rizza

From: **Chloé Yeung** chloe.yeung@gmail.com
Subject: Re: CDDis IMPORTANT agreement
Date: 31 March 2022 at 14:19
To: René Svensson svensson.nano@gmail.com
Cc: Costanza Montagna costanzamontagna@gmail.com, Monika Bayer mlbayer82@gmail.com, Monika Lucia Bayer Monika.Lucia.Bayer@regionh.dk, michaelkjaer@sund.ku.dk

CY

Dear Costanza,

I hereby confirm including Emiliano Maiani as an author of the new version of the paper by corresponding author Costanza Montagna, where I, Monika Bayer, am a co-author. Emiliano Maiani has significantly contributed to the additional experiment requested by the reviewers. I hereby agree with the change of authorship list to: Costanza Montagna, Rene Svensson, Monika Bayer, Salvatore Rizza, Emiliano Maiani, Chloé Yeung, Giuseppe Filomeni, Michael Kjaer.

Chloé Yeung

On Thu, 31 Mar 2022 at 2:08 pm, René Svensson <svensson.nano@gmail.com> wrote:

> Dear Costanza,
>
> I, Rene Svensson, hereby confirm including Emiliano Maiani as an author of the new version of the paper "Autophagy guards tendon homeostasis" by corresponding author Costanza Montagna. Emiliano Maiani has significantly contributed to the additional experiment requested by the reviewers. I hereby agree with the change of authorship list to: Costanza Montagna, Rene Svensson, Monika Bayer, Salvatore Rizza, Emiliano Maiani, Chloé Yeung, Giuseppe Filomeni, Michael Kjaer.
>
> Best regards,
> Rene Svensson
>
> ---
>
> Virus-free. www.avg.com
>
> On Thu, Mar 31, 2022 at 1:55 PM Monika Lucia Bayer <Monika.Lucia.Bayer@regionh.dk> wrote:
>
>> I hereby confirm including Emiliano Maiani as an author of the new version of the paper by corresponding author Costanza Montagna, where I, Monika Bayer, am a co-author. Emiliano Maiani has significantly contributed to the additional experiment requested by the reviewers. I hereby agree with the change of authorship list to: Costanza Montagna, Rene Svensson, Monika Bayer, Salvatore Rizza, Emiliano Maiani, Chloé Yeung, Giuseppe Filomeni, Michael Kjaer.
>>
>> Sincerely yours
>> /Monika Bayer
>>
>> -----Oprindelig meddelelse-----
>> Fra: Costanza Montagna <costanzamontagna@gmail.com>
>> Sendt: 31. marts 2022 12:17
>> Til: michaelkjaer@sund.ku.dk; svensson.nano@gmail.com; Monika Lucia Bayer <Monika.Lucia.Bayer@regionh.dk>; Monika Bayer <mlbayer82@gmail.com>; Chloé Yeung <chloe.yeung@gmail.com>
>> Emne: CDDis IMPORTANT agreement
>>
>> Dear co-authors,
>>
>> I need a formal agreement from all of you because I have included in the new version a new author, Emiliano Maiani, who helped me in the further analysis of immunofluorescence, as requested by the reviewers.
>> If you can reply to this mail confirming that you agree with the change, I will proceed with the final submission.
>>
>> The previous author list was:
>>
>> Costanza Montagna, Rene Svensson, Monika Bayer, Salvatore Rizza, Chloè Yeung, Giuseppe Filomeni, Michael Kjaer.
>>
>> Now:
>>
>> Costanza Montagna, Rene Svensson, Monika Bayer, Salvatore Rizza, Emiliano Maiani, Chloé Yeung, Giuseppe Filomeni, Michael Kjaer.
>>
>> Thanks
>>
>> Warm regards
>>
>> Costanza
>>
>> ________________________________
>>
>> Region Hovedstaden anvender de personoplysninger, du giver os i forbindelse med din henvendelse. Du kan læse mere om formålet med anvendelsen samt dine rettigheder på vores hjemmeside: www.regionh.dk/persondatapolitik

From: **Giuseppe Filomeni** giufil@cancer.dk
Subject: Agree on authors' list change
Date: 31 March 2022 at 13:36
To: Costanza Montagna costanzamontagna@gmail.com

GF

Dear Costanza,
I agree with the change in authors' list.
The previous authors' list was:

Costanza Montagna, Rene Svensson, Monika Bayer, Salvatore Rizza, Chloè Yeung, Giuseppe Filomeni, Michael Kjaer.

Now:

Costanza Montagna, Rene Svensson, Monika Bayer, Salvatore Rizza, **Emiliano Maiani**, Chloé Yeung, Giuseppe Filomeni, Michael Kjaer.

Best regards,

Giuseppe Filomeni

Danish Cancer Society
Strandboulevarden 49
DK-2100 Copenhagen
Telephone: +45 35 25 74 02

Danish
Cancer Society

www.cancer.dk | Vores privatlivspolitik

From: **salvatore rizza** salvatoreri@gmail.com
Subject: Authors' list Change
Date: 31 March 2022 at 13:15
To: Costanza Montagna costanzamontagna@gmail.com

SR

Dear Costanza,
I agree with the change in authors' list.

The previous author list was:

Costanza Montagna, Rene Svensson, Monika Bayer, Salvatore Rizza, Chloè Yeung, Giuseppe Filomeni, Michael Kjaer.

Now:

Costanza Montagna, Rene Svensson, Monika Bayer, Salvatore Rizza, Emiliano Maiani, Chloé Yeung, Giuseppe Filomeni, Michael Kjaer.

Best regards,

Salvatore Rizza
