# Supplementary material for: Autophagy guards tendon homeostasis
Source: Cell Death Dis. 2022 Apr 23;13(4):402. doi: 10.1038/s41419-022-04824-7 (PMC9035152; doi:10.1038/s41419-022-04824-7)
Supplement: Supplementary file 2 — Supplementaries figure legends [file 41419_2022_4824_MOESM2_ESM.docx]

**Supplementary Material**

**Figure S1: Autophagy at the myotendinous junctions.**

(**A**) Histological analysis of human *gracilis* tendon stained with hematoxylin and eosin (HE). Scale bars = 10 μm. (**B**) Pearson’s and (**C**) Mander’s correlation coefficient for co-localization of PC1 and Atg12 in human *gracilis* tendons. M1: Fraction of Atg12 in PC1. M2: Fraction of PC1 in Atg12. (**D**) Histological analysis of murine *Achilles* tendon stained with HE. Scale bars = 10 μm. (**E**) Pearson’s and (**F**) Mander’s correlation coefficient for co-localization of PC1 and LC3B in murine Achilles tendons. M1: Fraction of LC3B in PC1. M2: Fraction of PC1 in LC3B. **(G)** Representative images of human *gracilis* tendon MTJs immunolabeled for LC3B (green), nuclei stained with DAPI  (blue) and analyzed by fluorescence microscopy. M, myofibers. T, tendons. (**I**) Histological analysis of huma *gracilis* MTJs stained with hematoxylin and eosin (H&E) stain. (**H**) Representative images of LC3B puncta (autophagosomes) in the tip of the myofibers connected to the tendon in murine Achilles myotendinous junctions (MTJs). M, myofibers. T, tendons. MTJs were immunolabeled for LC3B (green), nuclei stained with DAPI  (blue) and analyzed by fluorescence microscopy. The inset shows higher magnification of the boxed area. (**J**) Histological analysis of murine MTJ stained with picrosirius red stain.

**Figure S2: Time-course experiments for PC1 degradation induced by Torin 1.**

(**A**) Western blot analysis of PC1, LC3B in Control, Torin 1 treated cells for 2 and 4 h. GAPDH was used as a loading control. (**B**) Quantification of the normalized PC1 protein level. Data are representative of three independent experiments made with cells from three different human donors. **p < 0.01, unpaired t-test. (**C**) Western blot analysis of PC1, LC3B and SQSTM1/p62 in control and Torin 1 treated cells for 4 and 24 h. GAPDH was used as a loading control. (**D**) Quantification of the normalized PC1 protein level. Data are representative of three independent experiments made with cells from three different human donors. *p < 0.05, ***p < 0.001, unpaired t-test. (**E**) Western blot analysis of phospho mTor (Ser 2448) and mTor in Control, Torin 1 treated cells from 0 to 4 h. (**F**) Quantification of the normalized phospho-mTor protein level. Data are representative of three independent experiments made with cells from three different human donors. **p < 0.01, ***p < 0.001, unpaired t-test. (**G**) Western blot analysis of phospho-p70 S6 Kinase in Control, Torin 1 treated cells for 1 and 4 h. LDH was used as a loading control.

**Figure S3: Immunofluorescence analysis of human tendon cells under basal condition.**

(**A**) Human tendon cells under basal conditions were immunolabeled for PC1 (red), LC3B (green) and nuclei stained with DAPI (blue). (**B**) Pearson’s and (**C**) Mander’s correlation coefficient for co-localization of PC1 and COPII in control and siAtg7-treated cells. M1: Fraction of COPII in PC1. M2: Fraction of PC1 in COPII. **p < 0.01 , ***p < 0.001, ****p < 0.0001, unpaired t-test. (**D**) Human tendon cells under basal conditions were immunolabeled for PC1 (red), CANX (blue) and LC3B (green). (**E**) Mander’s correlation coefficient for co-localization of PC1 and CANX . M1: Fraction of CANX in PC1. M2: Fraction of PC1 in CANX. (**F**) Mander’s correlation coefficient for co-localization of CANX and LC3B in control cells and treated with BafA1 for 16 h. M1: Fraction of CANX in LC3B. M2: Fraction of LC3B in CANX. ****p < 0.0001, unpaired t-test. (**G**) Control and siCANX-treated human tendon cells under basal conditions were immunolabeled for PC1 (red), LC3B (green) and nuclei stained with DAPI (blue). Cells were analyzed by confocal microscopy.  Scale bars = 10 μm. The insets show higher magnification and single color channels of the boxed area.

**Figure S4: PC1 accumulated as misfolded aggregates in BafA1-treated cells.**

(**A**) Western blot analysis of PC1, SQSTM1/p62 and LC3B in control and BafA1 treated cells for 4 and 24 h. Cell lysates were separated into soluble and insoluble fractions. GAPDH was used as a loading control. Quantification of the normalized PC1 protein level in soluble (**B**) and insoluble fractions (**C**). Data are representative of three independent experiments made with cells from three different human donors. **p < 0.01, ***p < 0.001, ****p < 0.0001, unpaired t-test.

**Figure S5: Western blot and gene expression analysis in 3D tissue-engineered tendons.**

(**A**) Western blot analysis of LC3B and SQSTM1/p62 in baseline, control and Torin1 3D tissue-engineered tendons. (**B**) Quantification of the normalized SQSTM1/p62 protein level and LC3BII/LC3BI ratio. Data are representative of three independent experiments made with cells from three different human donors. **p < 0.01, ***p < 0.001, ****p < 0.0001, unpaired t-test.

(**C**) p62/SQSTM1 and LC3B and collagens (**D**) genes expression in baseline, control and Torin1 samples. Data are representative of three independent experiments made with cells from three different human donors. All data are expressed as geometrical means ± geometrical SD, ns p > 0.05, paired t-test, Bonferroni corrected. (**E**) DNA content in baseline, control and Torin1 samples. n = 3 tissue donors, with a minimum of 8 tissue-engineered tendons per cell preparation for each treatment for each parameter measured, ns p > 0.05, unpaired t-test. Gene expressions of several ECM (**F**) and fibrillogenesis (**G**) components and in Baseline, Control and Torin1 samples. Data are representative of three independent experiments made with cells from three different human donors. All data are expressed as geometrical means ± geometrical SD, **p < 0.01, paired t-test, Bonferroni corrected.
