# Supplementary figures and images for: Autophagy guards tendon homeostasis
Source: Cell Death Dis. 2022 Apr 23;13(4):402. doi: 10.1038/s41419-022-04824-7 (PMC9035152; doi:10.1038/s41419-022-04824-7)

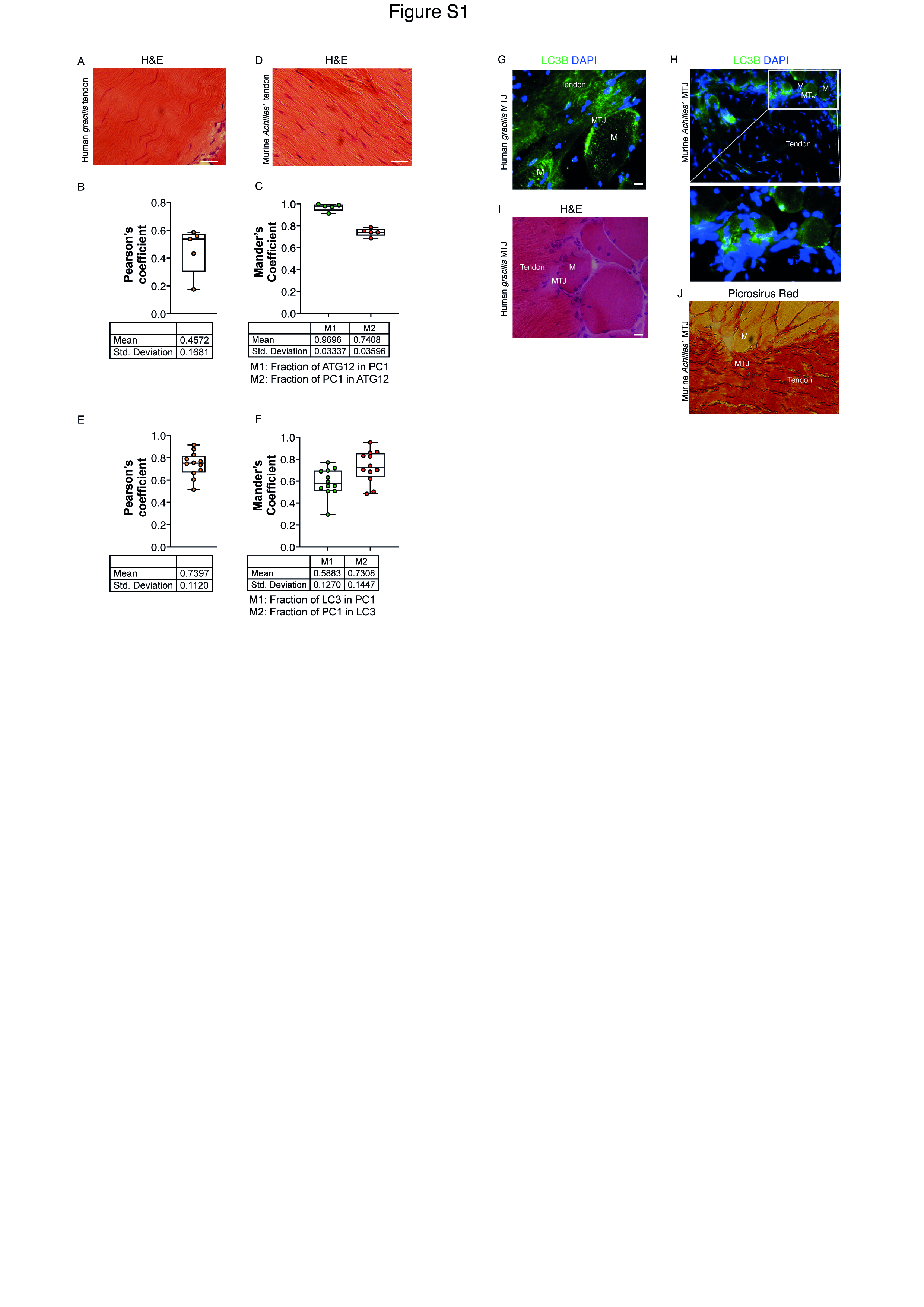

Supplement: Supplementary file 3 — Figure S1 [file 41419_2022_4824_MOESM3_ESM.tif]

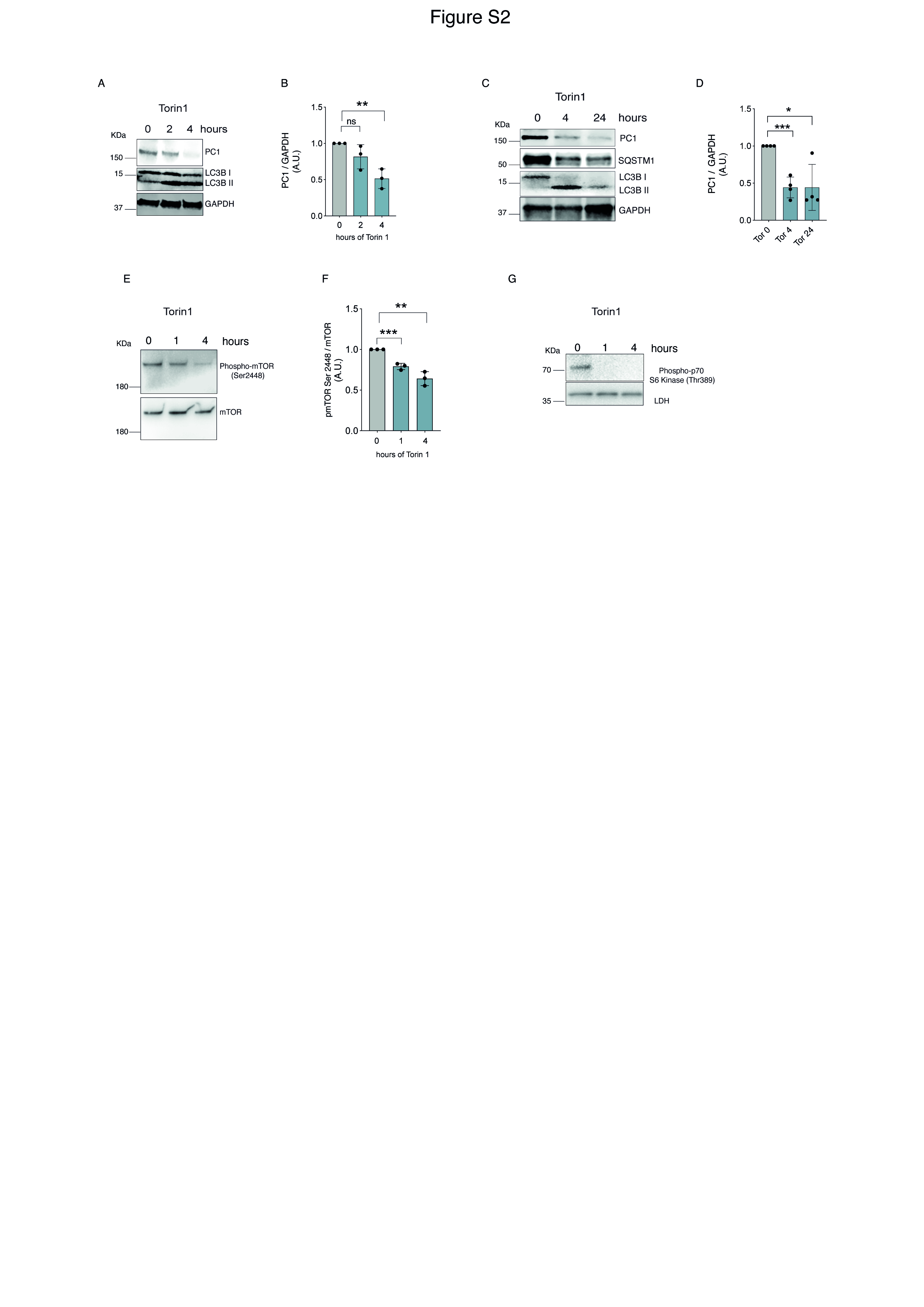

Supplement: Supplementary file 4 — Figure S2 [file 41419_2022_4824_MOESM4_ESM.tif]

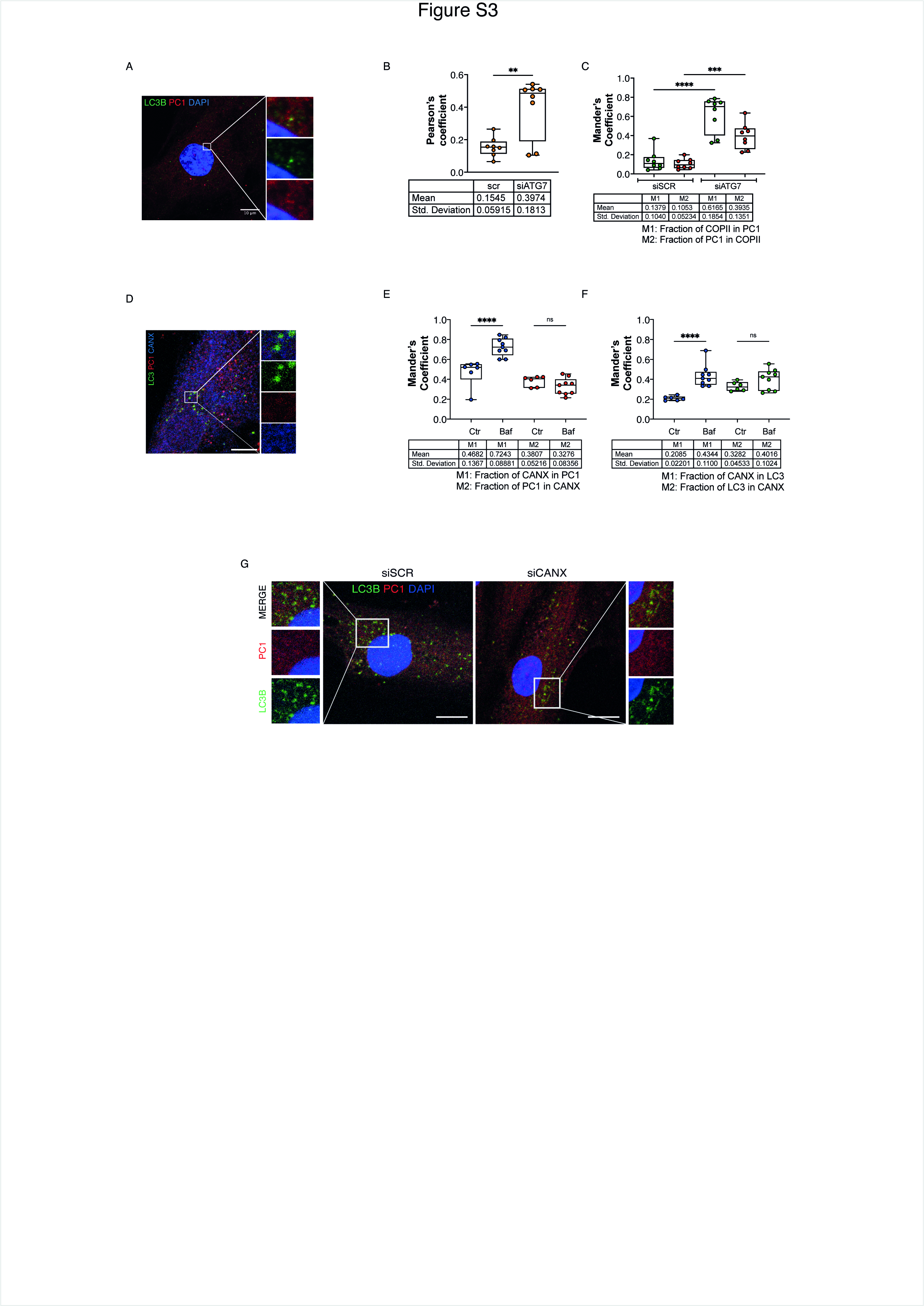

Supplement: Supplementary file 5 — Figure S3 [file 41419_2022_4824_MOESM5_ESM.tif]

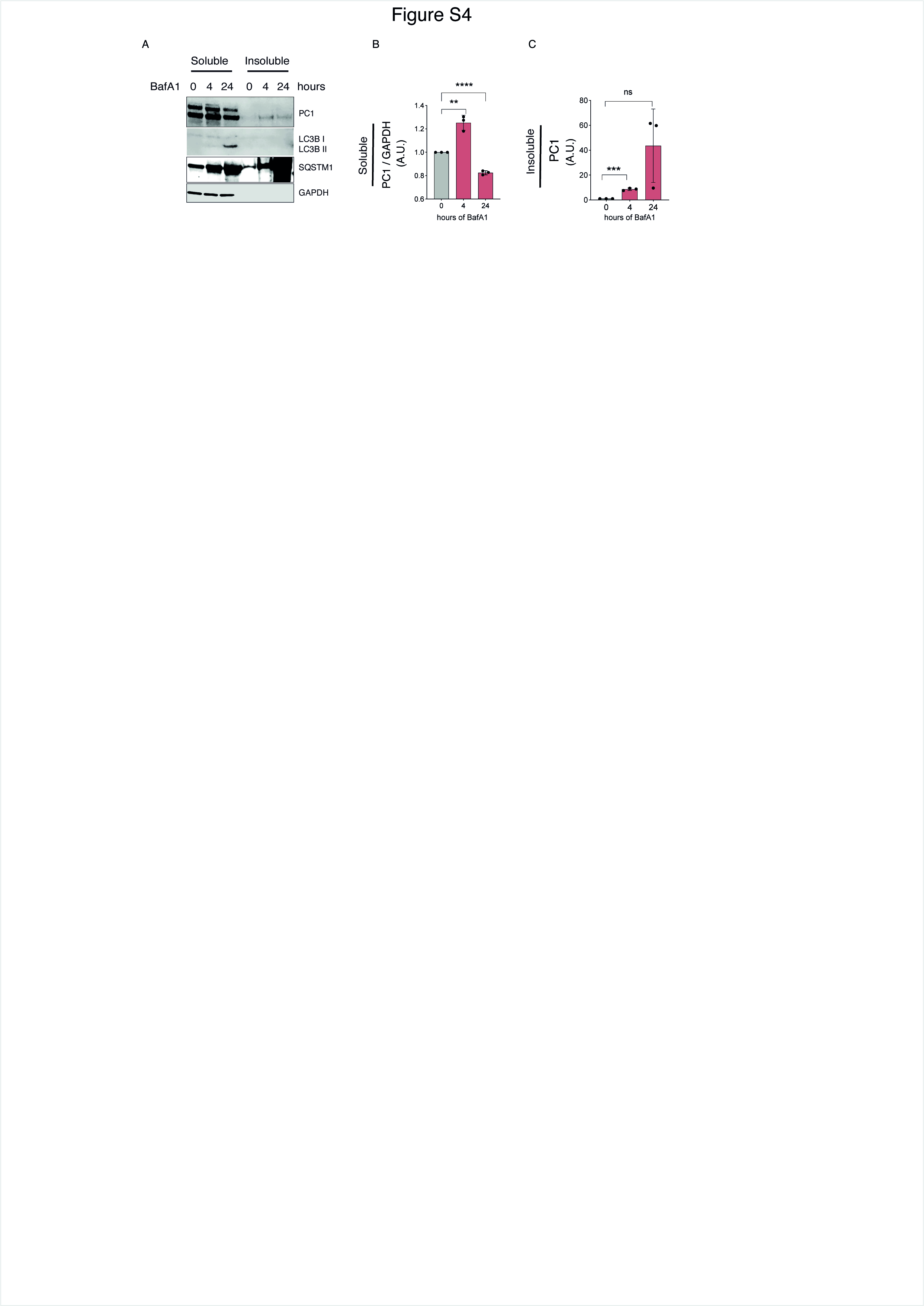

Supplement: Supplementary file 6 — Figure S4 [file 41419_2022_4824_MOESM6_ESM.tif]

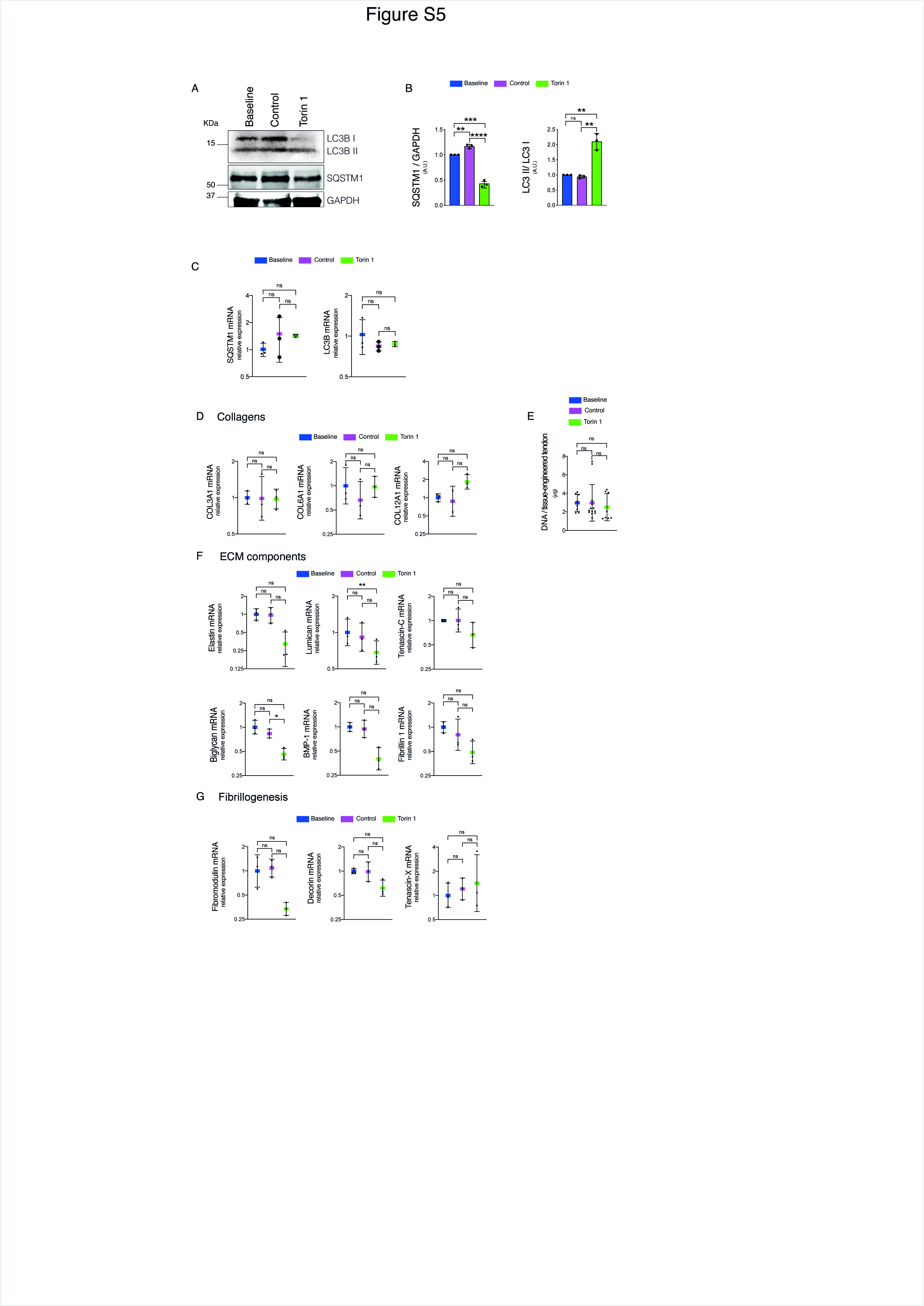

Supplement: Supplementary file 7 — Figure S5 [file 41419_2022_4824_MOESM7_ESM.tif]

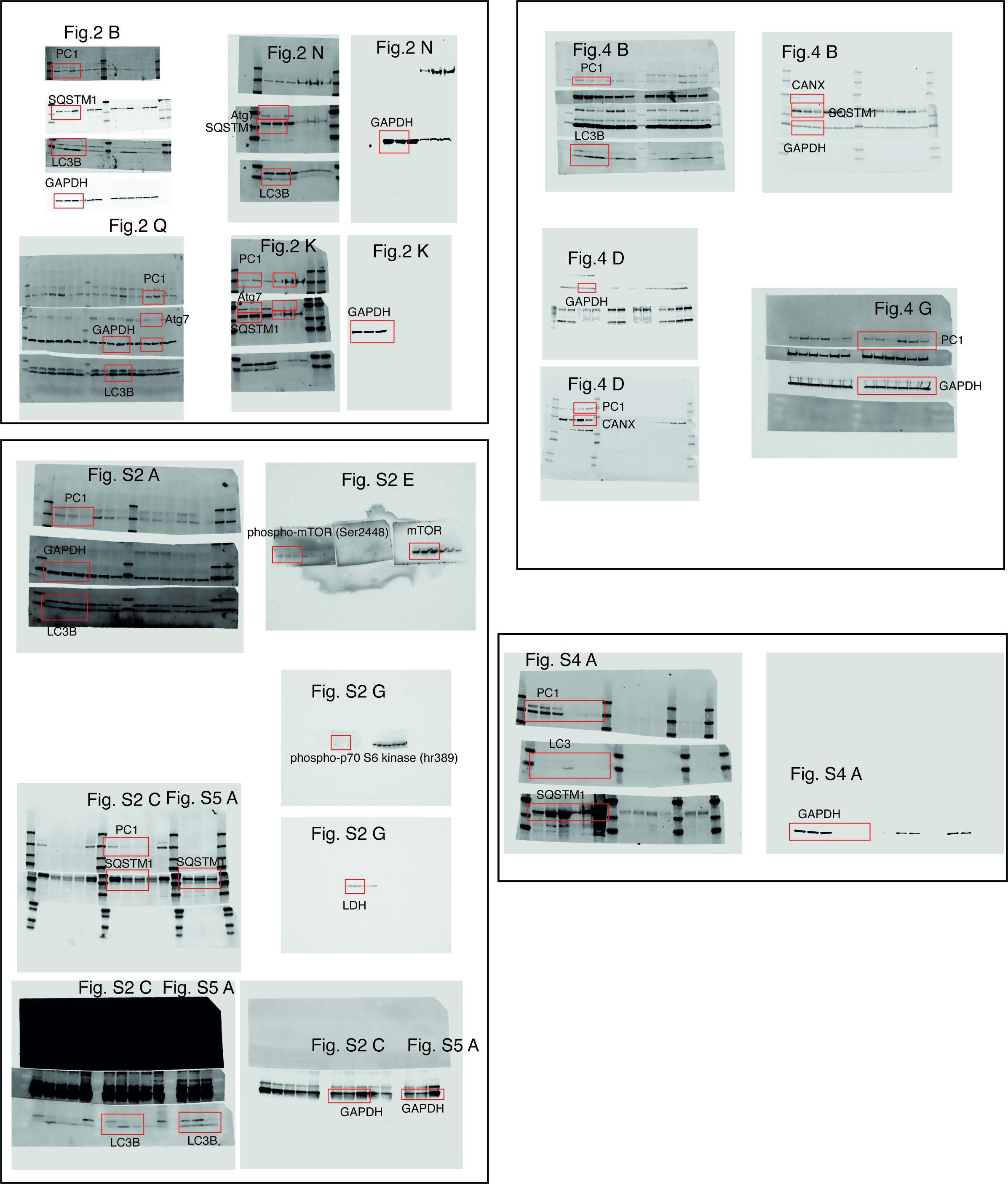

Supplement: Supplementary file 9 — Original Western Blots [file 41419_2022_4824_MOESM9_ESM.tif]
