# Supplementary material for: Autophagy guards tendon homeostasis
Source: Cell Death Dis. 2022 Apr 23;13(4):402. doi: 10.1038/s41419-022-04824-7 (PMC9035152; doi:10.1038/s41419-022-04824-7)
Supplement: Supplementary file 8 — Supplementary Table 1 [file 41419_2022_4824_MOESM8_ESM.docx]

Supplementary Table 1: Primers

| **mRNA Target** | **Sense Primer** | **Anti-sense primer** | **GenBank ID** |
| --- | --- | --- | --- |
| **Collagen I (COL1A1)** | GGCAACAGCCGCTTCACCTAC | GCGGGAGGACTTGGTGGTTTT | NM_000088.3 |
| **Collagen III (COL3A1)** | CACGGAAACACTGGTGGACAGATT | ATGCCAGCTGCACATCAAGGAC | NM_000090.3 |
| **Collagen VI (COL6A1)** | CACACCGCTCAACGTGCTCTG | GCTGGTCTGAGCCTGGGATGAA | NM_001848.2 |
| **Collagen XII (COL12A1)** | CCCAGGTCCTCCTGGATACTGTGA | GCAGCACTGGCGACTTAGAAAATGT | NM_080645.2 |
| **Collagen XIV (COL14A1)** | AGCATGGGACCGCAAGGC | GACGCGCCACTGATCTCACC | NM_021110.3 |
| **Elastin (ELN)** | GGCTTCGGATTGTCTCCCATTTT | CCAACGTTGATGAGGTCGTGAG | NM_000501.4 |
| **Lumican (LUM)** | CCCTGGTTGAGCTGGATCTGTC | CCAGGATCTTGCAGAAGCTCTTTATGT | NM_002345.4 |
| **Tenascin-C (TNC)** | CAGCCAAGATCCAGGCACTCAA | GTCCTTGGGGAAGGGGTACAGG | NM_002160.4 |
| **Biglycan (BCN)** | AGGCCAAGCTGACTGGCATCC | TGGCCTGGATTTTGTTGTGGTC | NM_001711.6 |
| **Bone Morphogenic Protein 1 (BMP1)** | CAGACGGCACACAGCTCGTAAGT | CAGACGGCACACAGCTCGTAAGT | NM_001199.4 |
| **Fibrillin 1 (FBN1)** | CGCTGCAATCATGGTTTCATCCTTT | ATTCCCATTTCCACTTGCACATTC | NM_000138.5 |
| **Fibromodulin (FMOD)** | CAGTCAACACCAACCTGGAGAACC | TGCAGAAGCTGCTGATGGAGAA | NM_002023.5 |
| **Decorin (DCN)** | GGTGGGCTGGCAGAGCATAAGT | TGTCCAGGTGGGCAGAAGTCA | NM_001920.5 |
| **Tenascin-X (TNXB)** | GGAGGACTATGCCCATGGTTTTG | CGCATGGAGTAGTCACCTGCCTGT | NM_019105.8 |
| **Glyceraldehyde-3-dehydrogenase (GAPDH)** | CCTCCTGCACCACCAACTGCTT | GAGGGGCCATCCACAGTCTTCT | NM_002046.5 |
| **Ribosomal protein, large, P0 (RPLP0)** | GGAAACTCTGCATTCTCGCTTCCT | CCAGGACTCGTTTGTACCCGTTG | NM_001002.3 |
| **Beta-2-microglobulin (B2M)** | GCTGTGCTCGCGCTACTCTCTCT | TCTGCTGGATGACGTGAGTAAACCT | NM_004048.4 |
| **Ribosomal Protein S26 (RPS26)** | AACACCCCCACCCCGATTTAGAC | GAACTCAGCTCCTTACATGGGCTTT | NM_001029.5 |
| **Cyclophilin A (PPIA)** | TGCAGACAAGGTCCCAAAGACAG | TGAAAGCAGGAACCCTTATAACCA | NM_021130.5 |
| **MAP1LC3B/LC3B** | CAGATCCCTGCACCATGCCGT | TTGGTTGGATGCTGCTCTCGAAT | NM_022818.5 |
| **SQSTM1/p62** | TGAAGAACGTTGGGGAGAGTGTG | GGCTTCTTTTCCCTCCGTGCT | NM_003900.5 |
